# Problem eating behaviors related to social factors and body weight in preschool children: A longitudinal study

**DOI:** 10.1186/1479-5868-4-9

**Published:** 2007-04-04

**Authors:** Lise Dubois, Anna Farmer, Manon Girard, Kelly Peterson, Fabiola Tatone-Tokuda

**Affiliations:** 1 Department of Epidemiology and Community Medicine, Faculty of Medicine, Institute of Population Health, University of Ottawa, Ottawa, Canada; 2 Institute of Population Health, University of Ottawa, Ottawa, Canada

## Abstract

**Background:**

Despite the increasing prevalence of overweight/obesity and its association to eating patterns in adolescents and adults, little is known about the relationship between problematic eating behaviours and body weight in the preschool years within the context of various social factors. This research aims to analyze the relationship between social factors, mothers' perceptions of their child's eating behaviour (picky eating and overeating), and body weight in preschool years, in a population-based cohort of preschoolers from Québec (Canada).

**Methods:**

Analyses were performed on 1498 children from the Longitudinal Study of Child Development in Québec, a representative sample of children born in 1998 in the Canadian province of Québec. Eating behaviours (picky eating and overeating) were derived from questionnaires at 2.5, 3.5, and 4.5 years of age. BMI was calculated from children's measured height and weight at 4.5 years. Children's sex and birth weight, mothers' age, immigrant status, smoking status during pregnancy, and education level, family type, annual household income and income sufficiency, the number of overweight/obese parents, children's day-care attendance, and food insufficiency were part of the analysis. Multivariate logistic regressions were used to determine odds ratios for different body weight profiles (underweight, normal weight, at risk of overweight, overweight), and one-way analysis-of-variances (ANOVA) allowed for group comparisons of means.

**Results:**

The proportion of children reported for each eating behaviour category remained quite stable across the years studied. Picky eating and overeating related to body weight among 4.5-year-old children, even when social and parental factors were accounted for in multivariate analysis. Picky eaters were twice as likely to be underweight at 4.5 years as children who were never picky eaters. Adjusted odds ratios revealed overeaters were 6 times more likely to be overweight at 4.5 years than were children who were never overeaters.

**Conclusion:**

Given the association between eating behaviours and bodyweight among 4.5-year-old children, particularly among those from less educated, lower income families and younger mothers, health professionals should target parents of children at risk of overweight/obesity and underweight with focussed messages and strategies for the management of emerging problematic eating behaviours.

## Background

An increase in prevalence of overweight among children and adolescents has been observed in many countries worldwide [1]. In 2004, 26% of Canadian children aged 2 to 17 were overweight, while 23% of children in Québec were overweight [2]. Childhood and adolescent overweight and obesity often track into adulthood and they have been shown to be predictive of child and adult morbidity and mortality [1]. Yet at the other end of the spectrum is childhood underweight which is associated with growth retardation, nutrient deficiencies and survival [3]. Although the prevalence of childhood underweight worldwide has decreased to 26%, only 1.6% of children in developed countries are underweight [4].

In light of increasing prevalence of obesity among children, there is growing interest in the eating patterns of children. Eating behaviours in childhood may vary on a continuum ranging from picky eating, irregular eating, overeating, and disinhibited or binge eating [5,6]. Picky eating at one extreme of the continuum is also known as 'neophobic', 'fussy eater', 'choosy', and 'problem eaters' across studies [7-9]. Although research on picky eating is limited, some studies observed that children who were picky eaters tend to: eat small meals, eat slowly, be less interested in food, acceptance of a limited number of foods, have an unwillingness to try new foods, have a limited intake of vegetables and other foods, and exhibit strong food preferences [10-12]. Picky eating may cause concern for parents about adequacy of the child's diet and they are also more likely to pressure a child to eat if they perceive the child to be underweight [13,14].

At the other end of the eating behaviour continuum is overeating which is defined as consuming large amounts of food within a specified period of time [15]. The concept of overeating overlaps with binge eating and disinhibited eating, however most definitions of overeating across studies does not include a sense of loss of control over one's eating as do binge or disinhibited eating behaviours [16,17]. Two studies that examined overeating in children and adolescents did not assess loss of control over eating in children and adolescents [18,19]. In addition to eating large amounts of food, overeating is also associated with eating more rapidly than normal, eating until uncomfortable, and eating large amounts of food when not feeling physically hungry [20]. Although some studies have shown higher eating rate (bites/second) among obese adults compared to normal weight adults [21], such differences is less known in young children's eating behaviours.

There is increasing recognition that problematic eating behaviours that manifest in early childhood may be a precursor to maladaptive eating later in life. For example, Marchi and Cohen [22] found that picky eating is associated with anorexia nervosa in adolescence. Another study found that prolonged feeding difficulties in children can lead to weight loss or failure to gain weight [23]. On the other hand, overeating may be linked to overweight and the development of binge eating in adolescents [24]. Moreover, concerns about eating, weight and shape are more prevalent among overweight children than normal weight children [25].

Little is known about the prevalence of problematic eating behaviours in healthy, normally developed children and the extent to which these behaviours change over time in different social contexts and their effect on body weight. It was hypothesised that a greater proportion of children who are picky eaters have a BMI below normal (< 10^th ^percentile), while a greater proportion of children who are overeaters have BMI above normal (≥95^th ^percentile). It was also proposed that varying social contexts is related to a child's eating behaviour. The aim of this research is to analyze the relationship of social factors (including mother's age, immigrant status, educational level, smoking status during pregnancy, family type, annual household income, income sufficiency, day care attendance, food insufficiency), the mother's perception of the child's problematic eating behaviours (e.g., picky eating and overeating), and body weight in preschool years in a population-based cohort of preschoolers from Québec (Canada).

## Methods

The analyses were performed using data from the Longitudinal Study of Child Development in Québec (1998–2002) (LSCDQ), conducted by Santé Québec, a division of the Institute of Statistics of Québec (ISQ), and funded by the Ministry of Health and Social Services of Québec [26,27]. The study analyzed the role of familial and social factors on children's health and on cognitive and behavioural development. The study followed a representative sample (n = 2103, response rate = 85%) of the children born in 1998 in the Canadian province of Québec (total population over 7 million, with approximately 70,000 newborns per year). The representative sample was based on a random selection of children born in each of the public health geographic areas of the province of Québec over the year such that the seasonality effect was minimized. Twins and children with major diseases at birth were not part of the study. The children were first seen at 5 months (gestational age, adjusted preterm birth) and then once a year thereafter. The study was based on face-to-face interviews using computer-based and self-administered questionnaires addressed to children's mothers and fathers. Children's questionnaires, including the eating behaviour questionnaire at 2.5, 3.5, and 4.5 years, were answered by the most knowledgeable person about the child, which was generally the mother. From the 2103 infants at the first data collection, 1944 remained at follow-up at 4.5 years in 2002 (children's ages ranging from 44 to 56 months, mean age of 49 months, SD ± 3.12 months). At 4.5 years, height and weight were measured following a standardized protocol for 1549 children. The ISQ estimated that this sample of 4.5 year-olds was still representative of the same-age children living in the province of Québec in 2002 [28].

Prior to the analysis, data were weighted by a factor based on the inverse of the selection probability, the probability of non-response, the post-stratification rate, and the attrition rate, to ensure that the data were longitudinally representative of the same-age children in the population [29]. Among the 1549 children, 1498 (97% of the sample) were part of the analyses; those excluded were children with no BMI values (due to a missing weight and/or height value) and with no indicator for their eating behaviours. The impact of missing data was evaluated by conducting with-and-without analyses. Given that missing data did not have an impact on the results, they were excluded from the analyses. Ethical approval from the Ministry of Health Ethics Committee (comité d'éthique de Santé Québec) and consent from participants were obtained.

### Dietary methodology

We chose to analyse problematic eating behaviours in children as they relate to BMI for two reasons. First, at the beginning of this study, we performed qualitative interviews with nutritionists working with obese children in various hospitals in Québec; it was observed in their practice that certain feeding behaviours among some children were related to weight status. Second, preliminary analyses performed with this dataset indicated that there was an association with eating behaviours and the child's weight status. The eating behaviour questions in this study were adapted from the Avon Longitudinal Study of Parents and Children (ALSPAC) [30] posted on the ALSPAC Website [31]. The eating behaviour questionnaire was reviewed by an expert advisory group on nutrition comprised of academics, researchers, and practitioners [32]. To follow, the instrument was pre-tested with a sample of parents of preschool children (n = 150) not included in the survey [28]. Based on these results, some questions were modified slightly for clarity and comprehension [27]. For more details on instruments and methods, see the "I am, I'll be" Website from the *Institut de la Statistique du Québec *[33]. The terms used to classify eating behaviours were given post-priori for the purpose of the analyses. Similar methods for categorizing eating behaviours were used by Ziegler and others [11].

The measures for eating behaviours were derived from self-administered maternal questionnaires at ages of 2.5, 3.5, and 4.5 years. The mother's perceptions of the child's eating behaviours were grouped according to two problematic eating behaviours: picky eaters and overeaters. The items used to identify picky eaters and overeaters are presented in Table 1. Picky eaters were comprised of children who "always" ate a different meal from that eaten by other members of the family, those who "often" refused to eat the right food, and those who "often" refused to eat. Overeaters were defined as children who "often" overate and those who "sometimes" or "often" ate too fast. It should be noted that the two categories of eating behaviours were not mutually exclusive and that a child can manifest more than one type of behaviour. The eating behaviours were analyzed separately for each time point (at 2.5, 3.5, and 4.5 years). Based on these results, an overall indicator for the 3-year period was constructed for identifying children's eating behaviours. Children were classified by scores obtained for eating behaviours. A sum of zero indicating a child as "never having the behaviour" throughout the period, while a sum of 3 indicated the child as "having the behaviour every year". Missing information for one year was discarded and the sum was based on the other years. Only a small number of children (n = 5) had missing information, yet it was possible to determine the overall behaviour from information on eating behaviours for the other two years. A preliminary analysis of the two eating behaviours indicated that only 1.6% to 2.2% of children exhibited both eating behaviours at each studied age indicating that these behaviours were mostly independent of each other. Multivariate analysis focussed on the overall eating behaviour indicator.

**Table 1:** Questions used for classifying children and proportion of children by eating behaviour at 2.5, 3.5 and 4.5 years

**Characteristic**	**Category**	**2.5 years**	**3.5 years**	**4.5 years**
***Behavior: Picky eater***		***14.0***	***16.9***	***15.9***

When she/he is at home with you for the main meal, how often does she/he eat a meal that is different from the other members of your family?	Always	**1.7**	**1.5**	**0.7**
	Almost always	3.7	2.5	2.5
	Sometimes	11.5	13.0	15.4
	Almost never	83.1	83.0	81.4
In general, does she/he refuse to eat the right food?	Never	29.0	22.7	17.9
	Rarely	20.9	20.5	22.8
	Sometimes	37.8	43.2	45.2
	Often	**12.3**	**13.6**	**14.1**
In general, does she/he refuse to eat?	Never	25.1	29.1	32.1
	Rarely	34.5	33.1	36.4
	Sometimes	36.0	33.7	28.1
	Often	**4.4**	**4.1**	**3.4**

***Behavior : Overeater***		***22.2***	***18.9***	***23.4***

In general, does she/he overeat?	Never	72.5	72.5	67.1
	Rarely	16.8	17.7	20.9
	Sometimes	**9.1**	**8.0**	**9.8**
	Often	**1.6**	**1.8**	**2.2**
In general, does she/he eat too fast?	Never	68.3	71.1	67.2
	Rarely	16.3	16.1	17.6
	Sometimes	**12.2**	**10.2**	**12.1**
	Often	**3.2**	**2.6**	**3.1**

### Body Mass Index

Children's height and weight were measured at home by a trained nutritionist following a standardized protocol using a measuring tape, ruler and scale [28] when children were 4.5 years-old. The children were weighed without shoes and wore light clothing. These measures were used to derive children's BMI (weight(kg)/height(m)^2^). Children were classified according to the US CDC sex- and age- specific growth charts [34], such that the category 'overweight' was defined as having a BMI at or above the 95th percentile, 'at risk of overweight' included children in the 85–94.9^th ^percentile, 'normal weight' encompassed children in the 10–84.9^th ^percentile, and the category 'underweight' included children below the 10^th ^percentile. These CDC cut-off's were used to analyse the gradient in associations present across weight categories.

### Predictor variables

Various factors related to eating behaviours and overweight were identified in a literature review and through consultation with experts. Variables pertaining to the child's characteristics included in the study are: children's sex and birth weight (less than 2500 g, 2500–4000 g, more than 4000 g). Maternal characteristics analyzed in the present study include: mother's age (less than 25 y, 25–29 y, 30–34 y, 35 y or more), immigrant status (immigrant or non-immigrant) education level (no high school diploma, high school diploma, college diploma, university diploma), and smoking status during pregnancy (smoker or non-smoker). Mothers were classified as immigrant and non-immigrant according to whether they were Canadian-born (non-immigrant) or immigrated from Europe or from non-European countries (immigrant).

Family characteristics selected for analysis include: family type (two-parent family, single-parent family); annual household income (< $20,000, $20–39,999, $40–59,999, >= $60,000); income sufficiency (sufficient or insufficient); the number of overweight/obese parents (zero, one, or two); children's day-care attendance (not in day-care or being taken care of at home, being taken care of at someone else's home, and in a day care centre); and food insufficiency (had not known food insufficiency, had known food insufficiency, from 0–18 months and-or from 3.5 to 4.5 years). The food insufficiency indicator used in this study was comprised of one question derived from previous work conducted on this cohort of children [35]. Participants were asked to respond to the question: "In the past 12 months, has a member of your family ever experienced being hungry because the family had run out of food or money to buy food?" with the following responses: "Yes, regularly once a month"; "Yes, more than once a month"; "Yes, certain months only"; "Yes, occasionally but not regularly"; or, "No". If any of the "Yes" categories were selected for the response, then participants were classified as "had known food insufficiency".

Sufficiency of income is determined by low-income cut-offs set by Statistics Canada for the reference year 1997 (1992 Baseline). It accounts for household income, the size of the household and the size of the residence area. Statistics Canada's low-income cut-offs (LICOs) are income thresholds at which a family would typically spend 20% more of its income than the average family on the necessities of food, shelter and clothing [36]. Families are classified as having 'sufficient income' when the household income is above the low-income threshold determined by Statistics Canada. When income is between 60% and 90% of the low-income threshold, households are classified as having 'insufficient income'; income levels below 60% of the low-income threshold are considered as 'very insufficient' [36]. Although LICOs are widely used, they do not measure poverty. Unlike the US, Canada does not have a measure of poverty. For example, low-income thresholds range from $20,047 for a family of four living in a rural area to $30,576 for similar families living in large cities [36].

The number of overweight/obese parents (based on BMI's) was derived from self-reported heights and weights obtained from each biological parent when the children were 1.5 years old. If both parents had a BMI of > = 25 then the number of overweight/obese was 2; if only one had a BMI of > = 25 then the variable was 1; and, if none of the parents had a BMI> = 25 then the variable was 0. A lot of effort was put to obtain the information for both parents. As a result, less than 3% of the sample had missing information for weight/height on at least one parent; for these cases, a missing value was attributed for this variable. The analyses were done including the missing category and this category was not found to have an impact on any analysis.

### Statistical analyses

Statistical analyses were done with SAS (version 8.2). All variables were treated as categorical variables except for BMI, the dependant variable, which was analyzed as both a continuous and a categorical variable. The adjustment variables includes the child's (sex, birthweight, day care attendance, food insecurity status), the mother's (age group, immigrant status, education, smoking status during pregnancy), and the family's (type, household income, number of obese parent) characteristic. The independent variables are the eating behaviours (picky eater and overeater). The dependant variable is children's BMI.

The analysis of the effect of the eating behaviours on the four BMI categories was done in three steps. First, bivariate analyses including the eating behaviours and the BMI as dependant variable were conducted using a chi-square test on contingency tables. Each behaviour was analyzed separately. As a second step, the eating behaviours were then included simultaneously in the model with the BMI as dependant. The analysis was performed using a polytomous regression with the second category of the BMI (10–84.9 th percentile) as the reference. Finally, the adjustments variables were included in the final model including the independent variables and the BMI as dependant. The final model was modified to include only the adjustment variables that were found to be significantly associated to BMI. However, preliminary analyses revealed that the adjustments retained for the final model were also associated to the eating behaviours in a bivariate analysis. Income and food insufficiency, when included in the final model, resulted in convergence problems due to zero-cell; they were removed from the final model. Food insufficiency was examined in a simpler model without income level and resulted in non-interpretable results, and therefore, it was not included in the final model. The small sample size of the food insufficient group contributed to some inconsistent results in the multivariate analysis. Mother's education, single-family parenting and income level were closely related and only the income level was kept in the final model since it had more impact on the BMI. Moreover, even though some variables were found to be associated to both the eating behaviour and the BMI, no interaction was found to be significant at the 0.05 significance level and they were not retained in the final model. Adjusted odds ratio (OR) estimates, as well as their confidence intervals, were then produced from the polytomous regression on the final model.

Adjusted means BMI were calculated by one-way analysis-of-variances (ANOVA) using the same adjustment variables identified in the final model from the analysis on the BMI in four categories. Post-hoc tests using Tukey's test were performed only when the overall F-test was significant. Weighted data were used in the analysis, and the significance level was set at 5% unless otherwise indicated.

## Results

Overall, almost one-third of the children (30%) were picky eaters and more than one-third (39%) were overeaters in their preschool years. Forty percent of the children were never reported by their mothers as having had any of these problematic eating behaviours from 2.5 to 4.5 years, while approximately 9% of the children were reported as having both these eating behaviours.

Table 1 represents the prevalence of different eating behaviours at 2.5, 3.5 and 4.5 years. The proportion of children reported as being picky eaters was quite stable in preschool years, ranging from 14% to 17%. The proportion of children reported as overeaters by their mothers ranged between 19% and 23% for the ages studied.

Table 2 presents a number of factors associated with each eating behaviour. Almost three-quarters (71%) of the children were never reported as being picky eaters, whereas 5.5% were picky eaters at 2.5, 3.5, and 4.5 years. The proportion of children who had this behaviour at the three studied ages was almost three times higher for children born with low birth weights (less than 2,500 grams) than children with higher birth weight (2500–4000, over 4000 grams) (14% vs. 5%). Income sufficiency was also associated with this behaviour. A greater proportion of children from insufficient-income families had been picky eaters at 3.5 years (22% vs. 16%) and at all three ages (9% vs. 5%) than had children from sufficient-income families (data not shown).

**Table 2:** Proportion of picky eaters and overeaters over all 3 ages by selected characteristics

			**Picky Eating**		**Overeating**	
**Characteristic**	**Category**	**%**	**Never reported as picky eater**	**Reported as picky eater at all 3 ages**	**Never reported as overeater**	**Reported as overeater at all 3 ages**

		***100.00***	***70.9***	***5.5***	***61.0***	***6.6***

Child's sex	Girl	49.51	72.1	4.8	64.7 *	4.4 *
	Boy	50.49	69.7	6.3	57.3	8.8
Birth weight	Less than 2500 g	4.03	69.0	14.2 *	52.2	11.3
	2500–4000 g	84.92	70.1	5.2	61.7	6.2
	More than 4000 g	11.04	77.0	5.1	58.8	7.7
Mother's age group	Less than 25 y	6.85	74.0	5.0	42.9 *	7.9
	25–29 y	23.14	69.8	3.9	59.0	8.0
	30–34 y	32.18	71.9	5.9	62.5	7.6
	35 y or more	37.82	70.4	6.4	64.3	4.7
Mother's immigrant status	Not immigrant	86.65	71.5	5.6	62.6 *	6.4
	Immigrant	13.35	68.3	5.6	51.4	7.9
Mother's education	No high school diploma	15.70	69.6	4.8	50.3 *	7.1
	High school diploma	21.87	67.7	6.7	58.2	7.0
	College diploma	34.93	68.9	5.3	66.1	7.0
	University diploma	27.50	77.0	5.5	62.9	5.5
Mother's smoking status during pregnancy	Did not smoke during pregnancy	74.97	71.6	5.7	61.7	6.0
	Smoked during pregnancy	25.03	68.8	5.1	58.7	8.5
Family type	Two-parent family	86.05	71.0	5.3	62.6 *	6.0 *
	Single-parent family	13.95	71.1	7.0	51.2	10.4
Annual household income	Less than $20,000	10.02	72.6	9.0	43.3 *	12.6 *
	$20,000–39,999	20.05	64.3	5.4	56.4	6.9
	$40,000–59,999	26.53	70.5	4.8	62.9	7.5
	$60,000 or more	43.40	73.7	5.3	66.1	4.7
Income sufficiency	Sufficient	80.30	71.5	4.8 *	64.5 *	5.9*
	Insufficient	19.70	68.4	8.8	47.4	9.5
Number of overweight/obese parents	0 parent	36.90	68.8	5.9	63.6	4.8 *
	1 parent	47.37	71.5	4.9	60.8	6.5
	2 parents	15.73	72.6	6.9	57.4	10.4
Day-care attendance	Not in day-care or being taken care of at home	30.58	73.8	6.3	57.4	5.8
	Being taken care of at someone else's home	35.37	71.9	4.8	64.4	6.4
	In a day-care centre	34.05	67.2	5.7	60.4	7.7
Food insufficiency in preschool years	Had never known food insufficiency	93.82	71.3	5.5	61.8*	6.2*
	Had known food insufficiency	6.18	65.6	5.4	48.6	12.5

* Statistically significant association between the characteristic and the dependent variable (p-value (chi-square)< = 0.05)

Sixty-one percent (61%) of the children had never been overeaters, whereas among 6.6% of the children the behaviour had persisted across all preschool years (Table 2). A greater proportion of boys than girls were overeaters at all of the studied ages. Single-parent family status, lower family income, income insufficiency, and having two parents who were overweight or obese were related to being classified as an overeater consistently over all three ages. The proportion of children classified as overeaters for all three ages was doubled in food insufficient families (12.5%) compared to food-sufficient families (6.2%). Children from young mothers (at 2.5 y), the ones from immigrant mothers (at 2.5 and 3.5 y) and from less educated mothers (at 2.5 y) were also more likely to be overeaters at these ages, compared to children from older, more educated and non-immigrant mothers (data *per *each age year not shown).

Table 3 presents the relationship between eating behaviours and BMI categories at 4.5 years. This table shows that a greater proportion of picky eaters were underweight (BMI below the 10^th ^percentile), and a lower proportion of picky eaters were at risk for overweight (BMI at 85–94.9^th ^percentile) compared to children never reported as picky eaters. A lower proportion of overeaters were underweight and a greater proportion of overeaters were at risk for overweight or were overweight (95^th ^percentile or more) at 4.5 years, compared to children who were never reported as overeaters. The proportion of overweight children increased with the number of years they were reported as overeaters, ranging from 5% if they were never reported with the behaviour, to 13% if they were reported one or two years, to 27% if they had this behaviour at every of the three studied ages.

**Table 3:** Proportion of children by BMI category^1 ^at 4.5 years and by eating behaviour

**Behaviour**	**Category**	**Underweight (< 10**^th^**percentile)**	**Normal weight (10–84.9**^th ^**percentile)**	**At risk for overweight (85–94.9**^th ^**percentile)**	**Overweight (> = 95**^th ^**percentile)**
***Total***		***15.2***	***64.4***	***11.3***	***9.0***

Picky eaters	Never reported as picky eater	13.2*	64.0	12.9*	9.9
	Reported as picky eater once or twice	18.3	67.3	7.6	6.9
	Reported as picky eater at all three ages	26.8	57.6	7.9	7.7
Overeaters	Never reported as overeater	18.2*	68.5	8.4*	4.9*
	Reported as overeater once or twice	11.3	60.1	15.5	13.1
	Reported as overeater at all three ages	6.8	47.9	18.3	27.1

^1 ^Percentiles from the BMI on the CDC USA Growth curves (age & sex specific curves)

* Statistically significant association between the characteristic and the dependant variable (p-value (chi-square) < = 0.05)

Multivariate analysis presented in table 4 indicates that when important factors for body weight were taken into consideration simultaneously (sex, birth weight, income level, parental overweight and obesity and mother's smoking status during pregnancy), being a picky eater at all three ages from 2.5 to 4.5 years increased the odds of being underweight at 4.5 years by 2.4 (95% CI 1.4–4.2), when compared with children who were never picky eaters. Compared with children who were never overeaters, having been an overeater at one or two of the ages studied more than doubled the odds of being at risk for overweight (OR 2.1, 95% CI 1.5–3.1) and almost tripled the odds of being overweight (OR 2.9, 95% CI 1.9–4.5), whereas having this behaviour at all three ages tripled the odds of being at risk for overweight (OR 3.2, 95% CI 1.7–6.1) and increased the odds of being overweight at 4.5 years by six times (OR 6.1, 95% CI 3.3–11.2).

**Table 4:** Adjusted odds^1,2 ^ratios (OR and 95% C.I.) for underweight, at risk for overweight, and overweight^3 ^children at 4.5 years by eating behaviour

**Behaviour**	**Category**	**Underweight (< 10**^th^**percentile)**	**At risk for overweight (85–94.9**^th^**percentile)**	**Overweight (> = 95**^th^**percentile)**
Picky eaters	Never reported as picky eater#	1	1	1
	Reported as picky eater once or twice	1.238 (0.877–1.747)	0.671 (0.427–1.054)	0.876 (0.534–1.438)
	Reported as picky eater at all 3 ages	2.415 (1.383–4.216)*	0.890 (0.376–2.106)	1.125 (0.458–2.766)
Overeaters	Never reported as overeater#	1	1	1
	Reported as overeater once or twice	0.815 (0.575–1.155)	2.149 (1.490–3.098)*	2.893 (1.880–4.452)*
	Reported as overeater at all 3 ages	0.471 (0.185–1.200)	3.249 (1.730–6.104)*	6.098 (3.308–11.240)*

^1 ^Adjusted for all of the included behaviours and for child sex and birth weight, income level, parental overweight/obesity, and mother's smoking status during pregnancy

^2 ^Dependant reference category = 10–84.9^th ^percentile

^3 ^Percentiles from BMI on the CDC USA Growth curves (age- & sex-specific curves)

* Significantly different from the reference category (p-value (type III – Wald chi-square test) < = 0.05)

An assessment of the relationship between the eating behaviours studied and adjusted mean BMI (adjusted for sex, birth weight, income level, parental overweight and obesity and mother's smoking status during pregnancy) revealed that, when other factors related to BMI were taken into consideration, mean BMI showed a significant increase (p = 0.05) with the number of years reported as an overeater (data not shown). Preschoolers reported as overeaters over all three ages studied and over one or two of the ages studied had a mean BMI of 17.3 and 16.4 respectively, in comparison to those never having been reported as an overeater (mean BMI = 15.9). Whereas mean BMI was significantly lower (p = 0.05) for preschoolers ever or always reported as picky eaters over the three studied ages (mean BMI = 16.4) in comparison to the children who had never had this behaviour between 2.5 and 4.5 years (mean BMI = 16.7).

## Discussion

The present study is one of few to report on children's eating behaviours in early childhood. A certain amount of problematic eating behaviours is to be expected in the toddler years as young children express their independence, however as young children transition to the preschool phase, problematic eating behaviours such as picky eating typically subside [9]. Our study indicates that eating behaviours are quite common and consistent across measured points even at young ages, with overeating being the most prevalent problematic eating behaviour we observed. The proportion of children reported by their mothers as overeaters ranged from 19% to 23% during the period when children were aged 2.5 to 4.5 years. Similarly, another longitudinal study of children conducted in England reported that 15% of mothers perceived their infants as overeating [30]. Whilst there are many differences in populations and definitions for eating behaviours across studies, it is difficult to establish the prevalence of problematic eating behaviours.

Few studies have further investigated the social determinants of problematic eating behaviours and body mass index of preschool children. The findings of the present study indicate that, overall, there is a significant association between characteristics of the child and family socioeconomic factors, the prevalence of problematic eating behaviours, and a child's bodyweight at 4.5 years. More specifically, one factor found to commonly relate to both problematic eating behaviours (picky eating and overeating) was family income insufficiency such that, children from families experiencing income insufficiency were more likely to report both problematic eating behaviours than those from income sufficient families.

Picky eaters were also more likely to have been born with a low birthweight (< 2500 grams). These results concur with another study [37]. On the other hand, children reported as 'overeating' were more likely to be male and showed many more associations with factors indicative of being raised in a family with low socioeconomic status, such as, being in a single-parent home, with low income, having experienced food insufficiency, and having two overweight or obese parents. A greater proportion of children from immigrant mothers were also reported as overeaters at 2.5 and 3.5 years compared to children from non-immigrant mothers.

The problem eating behaviours assessed in this study also demonstrated a significant association with children's bodyweight at 4.5 years of age. Children who were reported to be picky eaters were twice as likely to be underweight at 4.5 years, whereas, overeaters were six times more likely to be overweight at 4.5 years when these findings were adjusted for parental and social factors. For overeaters, the odds of being overweight were greater than the odds for being only at risk for overweight. Additionally, a possible stronger effect for stable behaviours over time was also suggested by the study results indicating that the proportion of overweight children rose as the number of years children were reported to be overeaters increased. These results concur with a recent study indicating that high maternal concern about an infant overeating or becoming overweight was associated with higher BMI at 5 years of age [38].

Studies revealing associations between low birthweight and underweight [39] also provide underlying support for the present studies findings of associations between low birthweight and picky eating, and picky eating with low BMI. Although some children who are born with low-birth weight may remain underweight throughout childhood, the underlying mechanisms for this relationship of birth weight and growth trajectories in childhood are poorly understood [40]. It is possible that for some low-birth weight children there is an underlying metabolic alteration that occurred in uterus that subsequently programs infants to becoming picky eaters, and that picky eating, in turn, subsequently keeps some children in the lower weight trajectory. However, this relationship requires further investigation to disentangle the possible biological and environmental causal pathways of these mechanisms. Another study reported that breast-fed infants seem to accept new foods more readily than formula fed infants [41]. We reported earlier that a larger disproportion of mothers who are lower income, less educated and younger mothers in this cohort do not breastfeed [42], and a similar profile of mothers reported picky eating. More research is required to obtain a thorough understanding of the effects of low birth weight, picky eating and environmental factors on body weight status of preschool children.

Overall, the finding that several factors indicative of a low socioeconomic status are associated with problematic eating behaviours, and these in turn associate with body mass index's in the extreme ranges (underweight and overweight) is interesting at many levels. Firstly, a larger proportion of children living in food insufficient households in our study were from families that had very insufficient income. Although it is challenging to separate specific effects of food insufficiency from income insufficiency given that food hardship frequently occurs in a context of poverty [43], research has shown a relationship between food insecurity and overweight in white girls aged 8–16 years [44] and preschool children [35]. The finding that these socioeconomic factors were associated with problematic eating behaviours in preschoolers, with corresponding longitudinal effects on BMI, may indicate that these eating behaviours explain part of the processes mediating these previous associations reported in the literature.

Second, although the literature on eating patterns of immigrant families is sparse, particularly for children, the higher prevalence of problematic eating behaviours observed in children from immigrant mothers is supported by the well established literature indicating that a larger proportion of immigrants, in particular refugees, in Canada are poorer, making poverty a possible confounder between the relationship between immigration and health [45]. Future research in areas related to food acculturation and coping strategies for feeding young children from different ethnic groups is warranted.

As in all studies, the present findings have strengths and limitations. Possible limitations of the study include the way in which the prevalence of problematic eating behaviours were based on mothers' subjective opinion of their child's eating behaviour; however, it appears that mothers' perceptions are accurate. In a previous publication on this cohort of children, energy intakes of overeaters were higher than picky eaters and the higher energy intakes were positively related to BMI [39]. Given that body weight is the best indicator of energy balance over time [46], these findings suggest although judgment of eating behaviours was subjective, mothers appear able to recognize problematic eating behaviours as reflected by children's weight. Additionally, although laboratory-based measures of eating behaviours may be considered more valid than self-reports, conducting observational studies on a large number of children would be difficult and may potentially influence children's eating behaviours. The small number of questions related to these indicators also creates little room for a wide range of validations. Others have reported similar challenges to using a more rigorous study design for this type of research [47]. For example, given the paucity of information on picky eating, it is not clear whether the concept is a parental notion and/or can be operationalised in terms of the child's behaviour [47-49]. Further investigation on behavioural validation, precursors and concomitants of eating behaviours is required for building a theoretical basis for future studies.

The strength of this study is its longitudinal design which is based on a population-based representative sample of children followed since birth. Second, both children's height and weight were objectively measured for calculating BMI measures. And finally, equally important is the high response rate (85%) of the questionnaires.

The family environment is key for the development of food preferences, patterns of food intake, eating styles, and the development of activity preferences and patterns that shape children's developing weight status [50-52]. It is well established that children's weight status may be shaped by family lifestyle behaviours. Some families have obesity-promoting characteristics, while others have obesity preventing characteristics that may explain differences in weight status over and above that explained by genetic susceptibility [53].

In summary, our study demonstrates that eating behaviours such as overeating may manifest very early in life and may be related to characteristics of the child and family, and the prevalence of overweight in children as young as 4.5 years-old. This is a particularly key finding since little is known about the first occurrence of problematic eating behaviours in young children and the relationship of these behaviours and body weight in preschool children. Further investigation regarding the specifics of various influences of eating behaviours on body weight is warranted.

## Conclusion

Given there is an association with different eating behaviours and bodyweight among 4.5 year-old children, particularly among those from families with lower socioeconomic characteristics, health professionals should be targeting parents of children at risk of overweight/obesity and underweight with focussed messages and strategies concerned with managing emerging eating behaviours including picky eating and overeating.

## Competing interests

The author(s) declare that they have no competing interests.

## Authors' contributions

LD is the director of the study (questionnaire development, data analysis, interpretation of the data, etc.). MG planned and performed the statistical data analysis. The AF and KP contributed to literature review, content analysis and writing of the manuscript. FT worked on the revision of the manuscript. All authors read and approved the final manuscript.
